# Validation of PECARN as a clinical decision rule for CT imaging of children with blunt abdominal trauma: a single centre retrospective cohort study

**DOI:** 10.1007/s00383-026-06664-4

**Published:** 2026-10-14

**Authors:** Jaime Tsz-wing Tsang, Adrian Chi-heng Fung, Carol Wing-yan Wong, Celestine Wing-Yin Ng, Jeff Wing-chiu Dai, Kenneth Kak-yuen Wong

**Affiliations:** 1 Department of Surgery, School of Clinical Medicine, Li Ka Shing Faculty of Medicine, The University of Hong Kong, Hong Kong, China; 2 Trauma Service, Queen Mary Hospital, Hong Kong, China

**Keywords:** Trauma, Children, Imaging, PECARN, Clinical rule

## Abstract

**Background:**

Blunt traumatic injuries are the leading cause of morbidity and mortality in children. Contrast enhanced computed tomography (CT) is the best imaging tool for screening patients at risk of blunt abdominal injury. Concerns existed on potential overuse and the radiation risks. We aim at evaluating paediatric use of trauma CT scan.

**Methods:**

Children with traumatic injuries who attended a tertiary trauma centre from January 2000 to December 2024 were included to evaluate the use of trauma CT scan and patterns of paediatric abdominal trauma. We excluded those with penetrating or isolated head injury. The Pediatric Emergency Care Applied Research Network (PECARN) algorithm was applied retrospectively with the aim of validating the use of PECARN.

**Results:**

Total of 1138 paediatic trauma admissions were identified during the study period and 70% (n 802) had blunt trauma. 483 patients were included after excluding patients with isolated head injury and missing data. 90 days trauma related mortality was 0.7% with one patient died within 24 hours of admission. Overall Injury Severity Score (ISS) was 3.52±5.95 which appeared to increase with age (rho 0.289, p <0.01).111 patients received CT scan, among which 35 are whole body CT. Significant findings were detected in 30% of these scans, 25 required intervention. Patients with higher trauma index and lower GCS, trauma call activation, older aged at presentation were more likely to receive a scan (p<0.001). No pathology was missed in the no CT group. PECARN validation was performed on 200 patients, There were no missed scans and pathologies in PECARN negative patients. Sensitivity was 100%, specificity was 96.5%, negative predictive value was 100%.

**Conclusion:**

A significant portion of children had negative trauma CT scan and did not need intervention. Use of clinical decision rule is warranted in identifying children at lower risk to minimize unnecessary imaging.

## Introduction

Trauma is the leading cause of death and morbidity among children and adolescents worldwide and raise public health concerns. Studies have shown that over 70% of paediatric trauma occurred by blunt mechanisms, with 10–20% involving intra-abdominal injuries (IAI) [1]. In Hong Kong, from 2006 to 2019, abdominal blunt injuries account for over 60% of paediatric blunt trauma with overall mean injury severity score (ISS) of 7.98 [2]. However, the severity and aetiologies of abdominal trauma in paediatric patients are highly heterogenous with many patients having relatively minor injuries that do not require surgical intervention. It is crucial to accurately identify and prioritize the most severely injured patients who need prompt surgical intervention while not to overuse computer tomography (CT) scans in patients with minor injuries.

Currently the usefulness of focused assessment with sonography (FAST) scan has limited evidence in children, with approximately 30% of paediatric IAI with free intra-abdominal fluid accumulation not shown on ultrasound scans [3]. Physicians still typically rely on CT scans during the initial diagnostic phase at the emergency department, supported by the American College of Surgeons Committee on Trauma (ACS COT), quoting that CT scan is still the best first-line imaging tool for patients with blunt abdominal trauma [4]. However, there is a lifetime risk of malignancy associated with radiation exposure, with large cohort studies reporting increased risks of leukemia and brain tumours after childhood exposure to CT radiation, with a positive dose-response relationship at cumulative doses of 50–60 mGys [5]. Studies have also shown that the inherent radiosensitivity of children puts them disproportionately at risk compared to adults.

Pediatric Emergency Care Applied Research Network (PECARN) is known for its application to paediatric head trauma, at the same time there is also a validated abdominal rule to identify IAIs. It involves seven variables and was validated and followed by some centres already [6]. It helps clinicians determine which children can safely avoid CT scans by identifying those who have none of the seven risk factors, including abdominal pain, abdominal wall trauma, seatbelt sign, vomiting, decreased breath sounds, thoracic wall trauma, or a GCS < 14. This study aims to examine the spectrum of paediatric trauma, evaluate the use of trauma CT scan in children in our locality, validate the use of PECARN and address the controversies of potential overuse of CT scans.

## Methods

### Study design

This retrospective, single-centre cohort study was performed through the prospectively collected trauma registry of a tertiary paediatric trauma centre concerning all children who attended an emergency department with various degrees of trauma. This retrospective cohort study was reported according to the Strengthening the Reporting of Observational Studies in Epidemiology (STROBE) guidelines for observational studies when appropriate [7].

### Setting, patients and data collection

Our centre is a tertiary referral centre for the specialist services of a network of hospitals. It is a key designated trauma centre on Hong Kong Island [8]. In general, trauma patients are either directly admitted from the scene of their injury to our centre’s emergency department or undergo secondary trauma diversion from a hospital in our network [9]. The network serves a population of about 0.6 million people. Data were retrieved from our centre’s prospectively collected, standardised trauma registry [8]. Chart reviews were conducted by a trained trauma coordinator to identify the majority of the data in the core set and extract supplementary data from electronic patient record system. Anonymised data were then entered into the registry by trained trauma registrars.

All children under 18 years of age with traumatic injuries who attended our tertiary trauma centre from January 2000 to December 2024 were included to evaluate the use of contrast CT scans in trauma setting and the patterns of paediatric abdominal trauma. The data in our analysis comprised patient demographics (including gender and age), hospital-processing information, classified activities, the need for trauma call activation, the types and severity of sustained trauma (defined using Injury Severity Scores [ISSs]), trauma mechanisms and whether and what type of CT scans were performed as well as their results. We excluded those with penetrating or isolated head injury. The Pediatric Emergency Care Applied Research Network (PECARN) algorithm was applied retrospectively with the aim of validation.

### Outcome measures

Primary outcomes of this study include the incidence of use of CT scan, presence of clinically-important intra-abdominal injuries (ciIAIs), predictive factors of use of CT scan in the emergency department, validation of PECARN using parameters including positive predictive value, negative predictive value, sensitivity and specificity. Clinically-important intra-abdominal injuries (ciIAIs) were defined in accordance with the original PECARN framework as any IAIs resulting in death, therapeutic laparotomy/interventional radiology, blood transfusion for abdominal hemorrhage, or intravenous fluid administration for two or more nights for abdominal trauma. Secondary outcomes were selected from the registry’s demographic and clinical data, including the types and severity of sustained injuries and morality rate.

### Statistical methods

All data were statistically analyzed and compared. The study’s statistical analyses were performed using the Statistical Package for the Social Sciences (SPSS; IBM, USA), Version 26. Descriptive statistics were presented as the number of units (n) and percentages (%). Data were expressed as means and standard deviations. Continuous variables were analyzed using Student’s t-test and an analysis of variance (ANOVA) test. Ordinal variables were analyzed using the Mann–Whitney U-test, and categorical variables were analyzed using the chi-square test. An unadjusted bivariate analysis was performed. A p-value < 0.05 was considered statistically significant.

## Results

A total of 1138 paediatric trauma admissions were identified during the study period and 70% (*n* = 802) had blunt trauma. 483 patients were included after excluding patients with isolated head injury and missing data. Incidence of IAI is 2%. Boys were more commonly injured with male-to-female ratio of 1.84:1. 90 days trauma related mortality was 0.7% with one patient died within 24 h of admission. Overall Injury Severity Score (ISS) was 3.52 ± 5.95 which appeared to increase with age (rho = 0.289, *p* < 0.01). Among the group, 111 patients received CT scan. Patients with higher trauma index (OR 11.89, CI 1.79, 79.1, *P* < 0.001), trauma call activation (OR 53.5, CI 6.9, 412, *P* < 0.001) and older age at presentation (OR 2.858, CI 1.85, 4.421, *P* < 0.001) were more likely to receive a scan. No pathology was missed in the no-CT group Table1.

483 patients were analyzed for PECARN validation, of which 38 (7.6%) had symptoms included in PECARN (PECARN-positive) while 420 (87%) were PECARN-negative, the rest involved incomplete data. Among the 38 PECARN-positive patients, 25 received contrast CT abdomen, 6 of them not receiving any CT scans and 7 of them received CT brain only. Among the 25 children who received contrast CT abdomen, 13 had clinically significant intra-abdominal injures (ciIAIs) which include liver, kidney or splenic lacerations, bowel injuries, etc. with 6 of them eventually requiring therapeutic laparotomy, 7 of them requiring interventional radiology. Whereas for the children who was PECARN-positive yet did not receive CT abdomen, there were no missed pathologies and were discharged uneventfully after observation in the general ward. For the 7 children who were PECARN-positive and received CT brain, they were PECARN-positive mainly because one of their presenting symptoms was vomiting. All of these children were ultimately found to have no abdominal pathologies, their CT brain scans were also negative.

On the other hand, among the total number of 420 PECARN-negative patients, there were still 22 children who received contrast CT abdomen and pelvis. All 420 children did not have any pathology at the end and no intervention was required. According to data in our locality, PECARN has a sensitivity of 100%, specificity was 97.2%. Its positive predictive value was 52%, negative predictive value was 100% Table2.

**Flowchart: Inclusion algorithm of patients**

**Figure:**
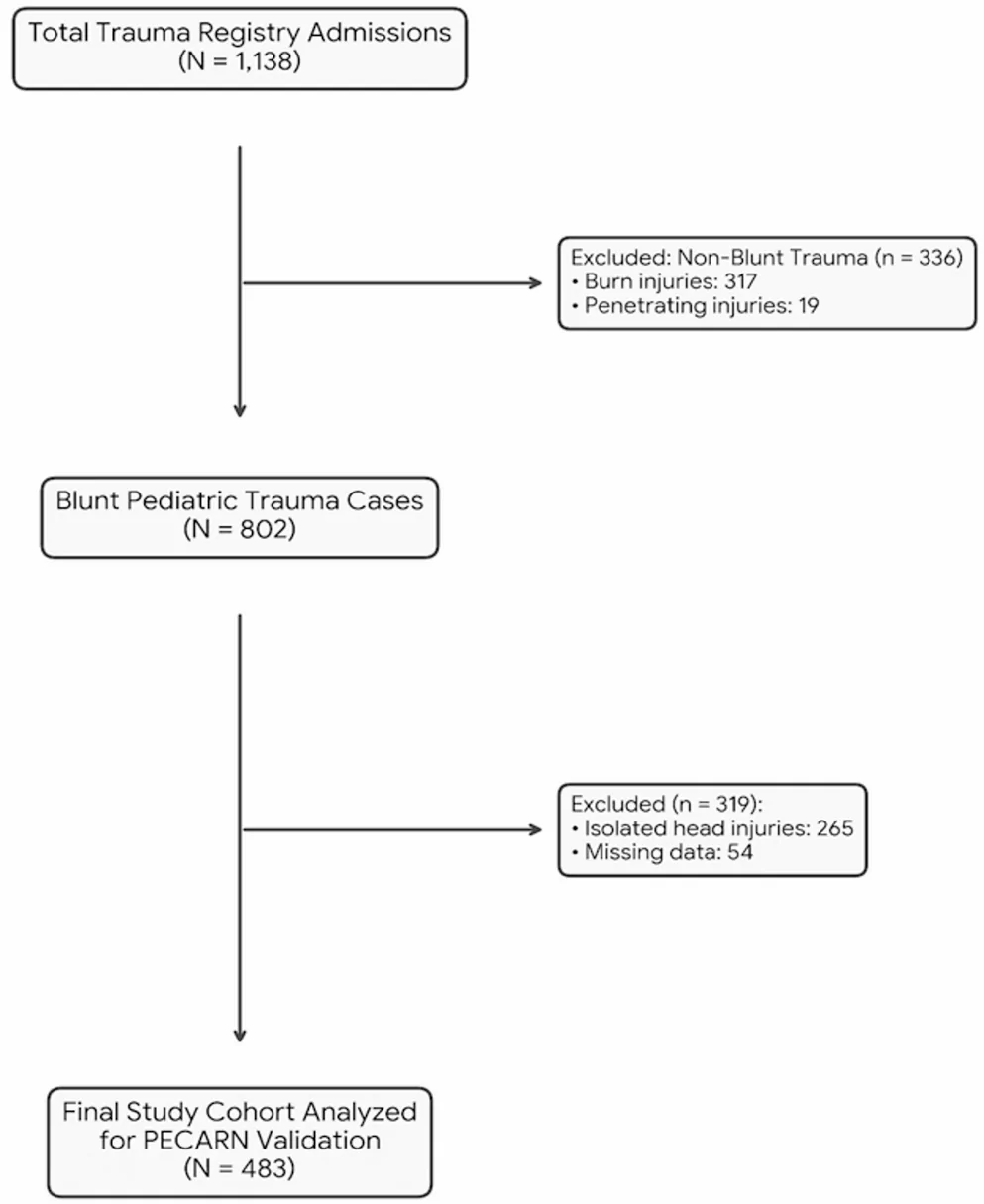

**Table 1:** Patient and clinical characteristics

	*N* (%)/mean/ratio	*p*
Occurrence of trauma	1138	
Gender (M: F)	1.84:1	0.205
Age group (years)		**< 0.001**
0–10	322 (66.7%)	
With CT done	53 (16.4%)	
11–18	161 (33.3%)	
With CT done	58 (36%)	
Trauma call activation	56 (11.6%)	**< 0.001**
With CT done	56 (100%)	
Injury severity score	3.52 ± 5.95	**< 0.001**
Mechanism of injury		
Blunt	802 (70.5%)	
Penetrating	19 (1.7%)	
Burn	317 (27.9%)	
Length of hospital stay (days)	4.2 ± 9.6	0.206
Length of ICU stay (days)	0.5 ± 3.0	0.855
Mortality		0.356
30-days	2	0.09
90-days	0.7	0.06

**Table 2:** Validation of PECARN

	Percentage (95% CI )
Sensitivity	100 (60.7, 100)
Specificity	97.2 (74.8, 97.2)
Positive predictive value	52 (52, 100)
Negative predictive value	100 (91.1, 100)

## Discussion

Paediatric blunt trauma is common worldwide, true intra-abdominal injuries (IAIs) account for a small proportion yet they could be fatal. In Japan, incidence of paediatric IAIs is 7.7% [10]. In the US, 90% of paediatric trauma are blunt trauma with IAIs account for around 5% of all cases [11]. However, IAIs are clinically important as they account for almost 25% of the most severe trauma cases with a significant mortality rate of 10% [12]. With such importance, it is crucial to promptly identify and diagnose IAIs in the emergency department. Yet, diagnostic accuracy in the paediatric population is often limited by various factors. For instance, children may have separation anxiety in hospital setting, uncontrolled temperament, delayed language development and limited intellectual capability, etc. Meanwhile, a prospective study conducted in US shows that the sensitivity of abdominal findings for IAI decreases as GCS score decreases [13]. In facing the challenges of diagnosing paediatric IAIs at the emergency department, imaging, i.e. contrast CT were widely ordered in trauma centres to reach accurate diagnosis (Tables 1, 2).

Contrast CT scans usage in paediatric trauma has been frequent. The rate of CT scan done quoted in our study is 13%, although lower than previous studies with rates of 25% [6], it is still crucial to cautiously avoid unnecessary radiation exposure as it has been found that accumulated doses of radiation increase the risks of brain tumours and leukaemia [5]. Meanwhile, children have longer life expectancy hence the adverse effects of radiation have more time to accumulate. Therefore, despite CT scan is widely available and easily accessible in many parts of the world, it is essential to set up and apply clinical decision rules to determine which group of paediatric trauma patients require CT scans and which do not at the emergency department.

The PECARN abdominal rule is a clinical prediction tool developed by Holmes et al. [6], risks of IAIs in paediatric blunt trauma patients were stratified after analyzing data from 20 emergency departments. The rule uses only medical history and physical examination findings as its variables, thus can be used by all clinicians regardless of location or resources. In the original study, the rule has a sensitivity of 97%, specificity of 42.5%, and negative likelihood ratio of 0.07 for predicting IAIs. In our study, applying PECARN to our local data retrospectively, it has 100% sensitivity and 97.2% specificity, which echoes with the high sensitivity of 82.8% and specificity of 78.7% quoted by Mahajan et al. [14]. The high sensitivity and specificity of PECARN show that it can correctly pick up IAIs that require intervention while at the same time exclude those with relatively minor injuries. It is also worth noticing that some patients receive CT brain alone even they are PECARN-positive, all of these patients had the symptom of vomiting only, with the chief complaint of headache and head injury in addition to blunt injury to the abdomen. It shows that inclusion of the variables that comprise the PECARN abdominal rules does not necessarily imply that an abdominal CT scan is required. PECARN is a valid clinical decision rule but should be used in conjunction with clinical judgement.

When placed in a regional context, our findings align closely with other validation efforts across Asia, which frequently demonstrate high sensitivities and negative predictive values. External validations of PECARN in cohorts across Japan demonstrate excellent safety profiles (high sensitivity), with a sensitivity of 100% and a specificity of 65.1% [10]. The same paper also reported FAST scan with AST/ALT having a sensitivity of 65.0% and a specificity of 88.7%, advocating the concurrent use of PECARN and these point-of-care tests in the emergency department, a strategy that warrants further prospective investigation in our locality.

The study has few key limitations. First, it is a retrospective study, it involves retrospective application of PECARN to existing medical records, with missing data, inaccurate non-electronic records in earlier years hence possible under-reporting of symptoms and clinical assessments. Second, it is conducted in a single centre, meaning the results may not be able to extrapolate to other setting due to difference in demographics and variable clinical assessment.

Third, our study yielded a substantially higher specificity (97.2%) compared to the original PECARN cohort (42.5%) and other western validation cohorts. This discrepancy suggests the presence of retrospective application bias and under-documentation. Because clinical signs (such as mild abdominal wall trauma or transient vomiting) may not have been meticulously charted in health records if they were deemed insignificant by the evaluating physician, these patients were classified as PECARN-negative in our retrospective review. This artifactually inflates the specificity within our sample. Furthermore, due to the low absolute number of positive IAI cases in our single-center cohort, the 95% confidence intervals for our diagnostic performance, particularly sensitivity, are wide. This lower statistical precision limits the robustness of our independent validation, meaning our 100% sensitivity should be interpreted with caution.

Meanwhile, the extended study period spanning 24 years (2000–2024) introduces potential chronological bias and methodological ambiguity due to evolutions in local trauma infrastructure and imaging capabilities. During the first decade of our study period, trauma imaging required the logistical transport of potentially unstable pediatric patients away from the resuscitation room to the main Diagnostic Radiology (DR) department. A major operational turning point occurred in September 2009 with the installation of a dedicated 16-slice CT scanner directly within the Accident & Emergency (A&E) Department, which vastly expanded 24-hour trauma evaluation capabilities without the risks of long-distance patient transfers. This was further accelerated in 2013 by the introduction of a rapid 320-slice CT scanner in the main DR department, aligning chronologically with the international publication of the PECARN abdominal prediction rules. These institutional milestones may naturally alter clinician thresholds for ordering CT scans across different eras, presenting a confounding variable in the long-term retrospective evaluation of CT utilization rates and PECARN’s historical applicability.

Meanwhile, we do not have data regarding their long-term follow-up hence future complications related to the trauma episode may not be taken into account. Also, whether the patient has symptoms and signs under the PECARN rule could vary among clinicians. Lastly, PECARN is a clinical decision rule, which does not involve point-of-care test or FAST ultrasound scan, which are evidenced effective tools in identifying paediatric IAIs in emergency setting. Despite these limitations, to our knowledge, our study is the first local study that validated PECARN as a sensitive and specific clinical decision rule. Future studies should therefore focus on prospectively validating PECARN as a clinical decision rule in combination with point-of-care test and FAST ultrasound scan in the aim of formulating tailored paediatric abdominal trauma protocol in our locality.

## Conclusion

A significant portion of children had negative trauma CT scan and did not need intervention. Use of clinical decision rule is warranted in identifying children at lower risk to minimize unnecessary imaging. In this single-centred paediatric trauma cohort, the PECARN abdominal rule demonstrated high sensitivity and specificity which demonstrates the valid use of it in detecting IAIs in our locality. Further prospective multi-center studies are needed to determine its integration in paediatric trauma algorithm in our locality.

## Data Availability

Data are available upon approval through a data sharing agreement due to privacy rules.
